# Risk Assessment and Prophylaxis of Venous Thromboembolism in Patients of Medical Ward of Northwest General Hospital and Research Center, Peshawar, Pakistan: A Quality Improvement Project

**DOI:** 10.7759/cureus.32811

**Published:** 2022-12-22

**Authors:** Muhammad Haris Shah, Muhammad Sajjad Khan, Shahzad Ahmad, Uzma Aftab, Aimal Khan, Wiqar Ahmad, Umar Iftikhar, Izaz Ahmad

**Affiliations:** 1 Department of Medicine, Northwest General Hospital and Research Center, Peshawar, PAK; 2 Department of Medicine, Rawalpindi Medical University, Rawalpindi, PAK; 3 Department of Medicine, Pak International Medical College, Peshawar, PAK

**Keywords:** prevention, vte prophylaxis, critical care and internal medicine, hospitalized patients, quality improvement and patient safety, venous thromboembolism (vte), clinical audit system

## Abstract

Background

Venous thromboembolism (VTE) is a condition that occurs when a blood clot forms in veins. Hospitalization increases the risk of VTE so timely risk assessment and adequate prophylaxis for VTE should be done to prevent this potentially fatal complication.

Local problem

Data from developing countries regarding VTE prophylaxis is scarce. VTE is a neglected area of research in Pakistan. So this closed-loop clinical audit was conducted to evaluate the VTE risk assessment and prophylaxis practices and to analyze the importance of educational intervention in improving the standard of care.

Patients and methods

We adopted the National Institute for Health and Care Excellence (NICE) guidelines for VTE prophylaxis as an audit standard. We collected data on a specially designed proforma by prospectively reviewing the hospital notes of patients in the Medical Ward of Northwest General Hospital and Research Center, Peshawar, Pakistan. Phase A included 60 patients and after educational intervention, Phase B was conducted with 90 patients.

Intervention

The results of Phase A were presented in the Clinicopathological Conference (CPC) meetings of the hospital. Healthcare workers were educated regarding the risks of VTE and the importance of timely prophylaxis. Posters were also displayed in the ward for highlighting the importance of VTE prophylaxis.

Results

In Phase A, only 5% of patients were risk assessed for VTE and of those eligible for prophylaxis only 22.2% received the prescription. Phase B showed a significant adherence to standard practices. In Phase B, 100% of patients were risk assessed for VTE and 75% received the prophylaxis.

Conclusion

There was poor compliance with standard VTE risk assessment and prophylaxis prescribing practices. However, a simple and effective educational intervention markedly improved patient care in terms of VTE strengthening the impact of clinical audits in the improvement of care.

## Introduction

Venous thromboembolism (VTE) is a disease entity characterized by the formation of blood clots in veins [1,2]. Its symptoms range from relatively less severe deep vein thrombosis to fatal pulmonary embolism [3,4]. There is no published epidemiological data regarding the incidence of VTE in Pakistan [5]. The incidence of hospital-associated VTE in lower/middle-income countries is 1.1/1000 as compared to 3.5/1000 in high-income countries as suggested by a retrospective data-based modeling study [6]. Anyone can suffer from VTE but certain conditions including major surgery, hospitalization, immobility, older age, chronic diseases, increased estrogen, family history, and some congenital conditions multiply the risk of VTE to many folds [4]. The risk of VTE is particularly increased in patients hospitalized with acute medical illness. The significant morbidity and mortality associated with VTE can be prevented by adequate interventions [2]. Risk assessment of hospitalized medical patients can be performed using Padua, IMPROVE, Kucher, and Intermountain scores followed by the use of appropriate pharmacological prophylaxis including low molecular weight heparin [7].

Researches have shown that despite the availability of adequate guidelines, the risk assessment and prophylaxis of VTE are under-practiced in developing countries [8]. Consequently, besides being adversely linked to patients’ health this negligence also imposes a significant financial burden on already struggling economies [3]. To identify the problem, we conducted a clinical audit to assess the VTE risk assessment and prophylaxis practices in the medical unit along with the extent of compliance under the standard guidelines.

Aims and objectives

We aimed to assess the VTE prophylaxis practices by reviewing the documentation done in terms of assessment of risk, indications, and contraindications to prophylaxis and prophylaxis prescription for patients hospitalized in the medical unit of Northwest General Hospital and Research Center, Peshawar, Pakistan. We also aimed to analyze the effectiveness of educational intervention by conducting a re-audit.

Audit criteria and standard

Current practice is compared to the National Institute for Health and Care Excellence (NICE) guidelines for VTE prophylaxis which states that all patients, on admission, should receive an assessment of VTE and bleeding risk using the clinical risk assessment criteria described in the national tool [9]. For practical purposes, VTE risk assessment and prophylaxis, done in at least 90% of patients, was kept as an audit standard.

## Materials and methods

Phase A of the closed-loop clinical audit was performed from 15 January 2022 to 15 February 2022 at the medical ward of Northwest General Hospital and Research Center, Peshawar, Pakistan. Department of Health VTE risk assessment tool designed by NICE was used for risk assessment of VTE [10]. Age more than 60 years, obesity (BMI>30kg/m^2^), previous history of VTE, active cancer, thrombophilia, chronic cardiovascular, respiratory and endocrine problems, acute myocardial infarction or ischemic stroke, and reduced mobility expected for >3 days for any reason were among the indications for thromboprophylaxis. However active bleeding, acute hemorrhagic stroke, blood pressure >230/120 mmHg, bleeding disorders, and use of anticoagulants were among the contraindications of thromboprophylaxis. Team members gathered the data by prospectively reviewing the medical notes of patients admitted during this time.

Results of this audit were shared in the Clinicopathological Conference (CPC) of the hospital on two occasions one month apart. All grades of doctors, interns, and nursing staff attended these meetings and were sensitized regarding the importance of VTE risk assessment and prophylaxis. With the consent of the Head of the Department of Medicine, documentation regarding the VTE risk assessment of all patients and prophylaxis prescription was made compulsory for the medical team of the concerned unit. Moreover, posters narrating the importance of VTE prophylaxis were also displayed in different sections of the ward. To close the audit loop, Phase B of the clinical audit was conducted from 15 June 2022 to 15 July 2022 in the same department.

## Results

Phase A

Data from 60 patients were collected to assess the current practice of VTE risk assessment and prophylaxis. Of these 37 (61.67%) were males while 23 (38.33%) were females. Twenty-one patients (35%) were below 50 years of age, 16 (26.67%) patients belonged to the 50-60 years age category, 17 patients (28.33%) were between 60 and 70 years, and six patients (10%) were in above 70 years age group. As per clinical notes, assessment for VTE risk was done only in three (5%) patients.

**Figure 1:**
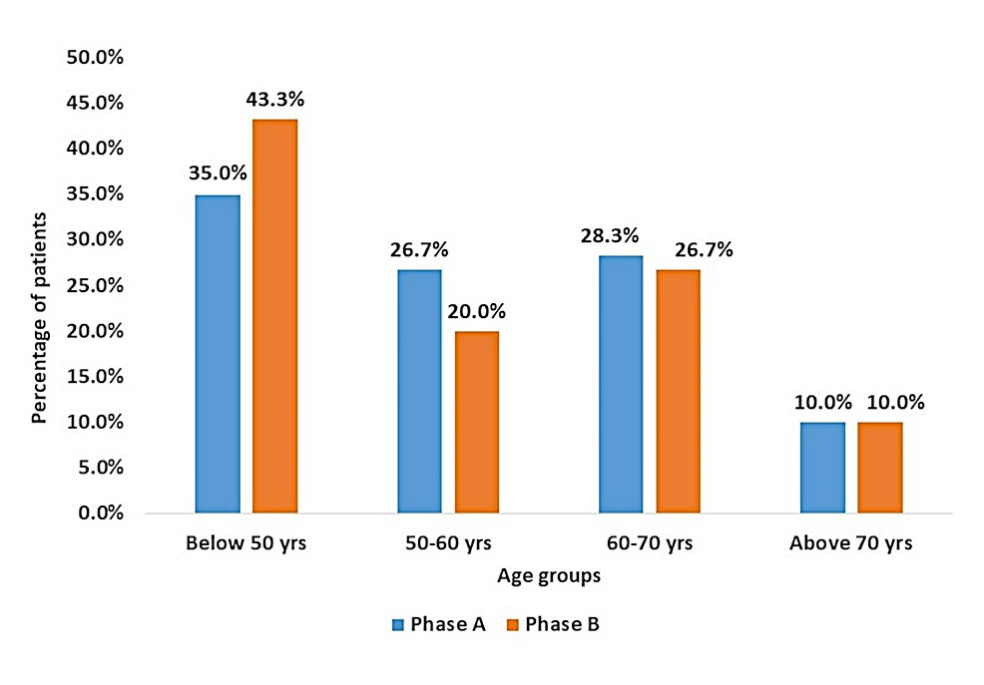
Age-wise distribution of patients in Phase A (audit) and Phase B (re-audit)

Among the total 60 patients, 30 (50%) had a clinical indication for VTE prophylaxis out of which three (10%) patients had a contraindication, high risk of bleeding being the major cause. Thus 27 (90%) patients out of 30 needed to have prophylaxis, of which only six (22.2%) patients received the prophylaxis prescription. All patients received drugs and doses as per the standard practice. Age-wise comparison of patients in Phase A with Phase B is depicted in Figure 1.

Phase B

Clinical notes of 90 patients in the same ward were analyzed. The study group comprises 48 (53.3%) females and 42 (46.67%) males. Age-wise distribution of patients in Phase B compared to Phase A is given in Figure 1.

**Figure 2:**
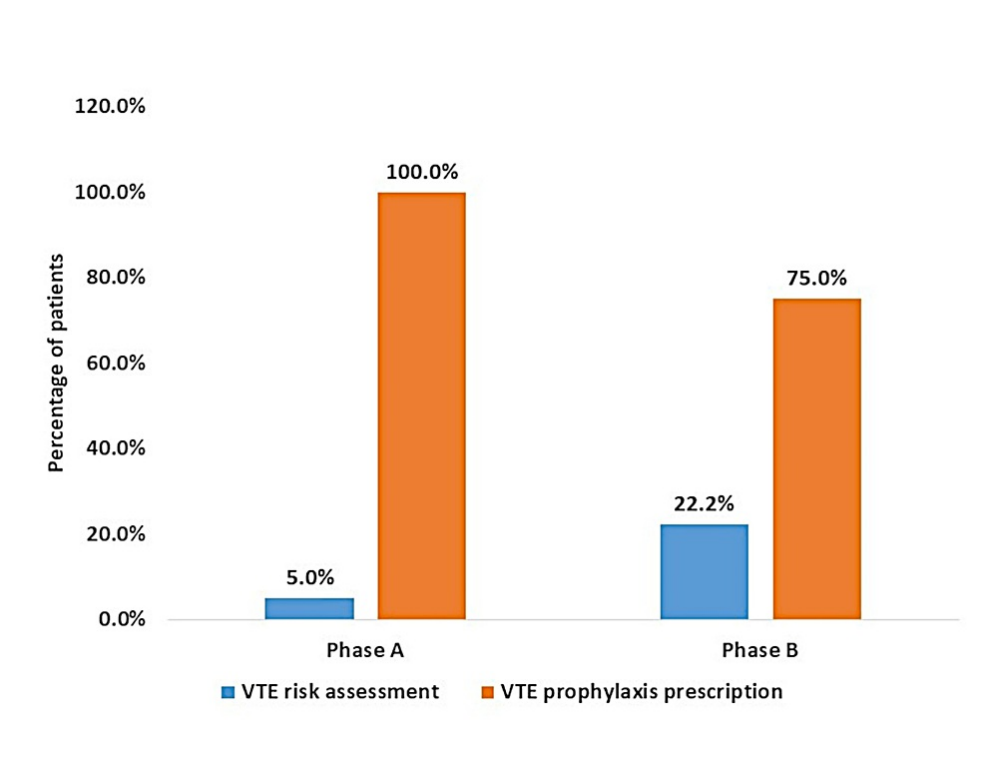
Comparison of VTE risk assessment and prophylaxis prescription between Phase A and Phase B VTE: venous thromboembolism

Evaluation of clinical notes revealed that risk assessment for VTE was done in all 90 (100%) patients. Among these, 24 (26.67%) patients had a clinical indication for VTE prophylaxis out of which three (12.5%) patients had a contraindication for VTE prophylaxis. Thus 21 (87.50%) patients out of 24 needed the VTE prophylaxis, out of them 18 (75%) patients were given the prophylaxis. Three (12.5%) patients who had an indication were not prescribed prophylaxis despite the absence of any contraindication. All patients received drugs and doses as per standard practice. Figure 2 depicts the comparison between the Phase A audit and Phase B re-audit in terms of the percentage of patients receiving the risk assessment for VTE and prescription of prophylaxis to highlight the impact of educational intervention.

## Discussion

VTE is not only a frequent cause of adverse prognosis in hospitalized patients but also contributes to more than 10% of hospital-related deaths [2,11]. Hospital-acquired VTE has a prevalence of 0.8-11% worldwide [8]. Well-researched and widely accepted guidelines are available for risk assessment and prophylaxis of VTE in hospitalized patients [2]. Very limited data is available from developing countries evaluating the adherence to standard guidelines regarding VTE risk assessment and prescription of appropriate prophylaxis [2]. In Pakistan VTE is a “neglected research agenda” and no national epidemiological data regarding the incidence and implementation of VTE prophylaxis is published. This could be attributed to a combination of a lack of research interest in this issue and a scarcity of necessary resources [5]. This closed-loop clinical audit was conducted using a specially designed proforma and it yielded some significant findings. Phase A of the audit reflected poor adherence to the standards of VTE prophylaxis where only 5% of patients were risk assessed for VTE. Similarly, among the patients eligible for VTE prophylaxis only 22.2% received it. These findings are consistent with a multicentre study conducted in Gaza which also depicted poor compliance regarding VTE prophylaxis [8]. In comparison with underdeveloped countries, the implementation of VTE prophylaxis is higher in developed countries [12]. This could be due to a lack of necessary supervision and a heavy workload relative to scarce resources in low-income countries. This negligence can also be attributed to a lack of uniform treatment guidelines and less clinical documentation due to the absence of electronic medical records.

Following the results of Phase A of the audit, the medical staff was sensitized regarding the need for risk assessment and prophylaxis of VTE. This was achieved by educational intervention and the compulsion of documentation. The effectiveness of this campaign was assessed by the re-audit Phase B which depicted some striking findings. Hundred percent of patients in the medical ward were risk assessed for VTE and of those requiring prophylaxis, 75% received it. This reflects the role of clinical audits in improving the quality of care provided to patients. Statistically significant improvement in VTE risk assessment after the educational intervention was also observed by McGoldrick in his closed-loop clinical audit [13].

Marked improvement in VTE risk assessment and prophylaxis observed after the educational intervention during the audit cycle reflects the main strength of this audit. Moreover, the recommendations made to the Department of Medicine based on this audit will further improve patient care in terms of prophylaxis of VTE. However, owing to the small sample size the results cannot be generalized. Based on the results of this audit, recommendations will be made to the concerned committee to include the VTE risk assessment and prophylaxis prescription in the drug kardex of the hospital. The effectiveness of this intervention can be assessed by further conducting a third cycle of the audit.

## Conclusions

VTE is a fatal yet preventable disease that poses serious threats to the life of patients and increases the financial burden on struggling economies. Although poor adherence to standard guidelines was observed in Phase A a simple educational intervention markedly improved the patient care related to VTE risk assessment and prophylaxis. This reflects the importance of conducting clinical audits in the quality improvement of clinical practice.
